# Adenocarcinoma and squamous cell carcinoma associated with gastric ulcers in alpacas

**DOI:** 10.1038/s41598-024-71079-x

**Published:** 2024-12-23

**Authors:** Saskia Neubert, Matthias Gerhard Wagener, Martin Ganter, Christina Puff

**Affiliations:** 1 Clinic for Swine and Small Ruminants, Forensic Medicine and Ambulatory Service, University of Veterinary Medicine Hannover, Foundation, 30173 Hannover, Germany; 2 Department of Pathology, University of Veterinary Medicine Hannover, Foundation, 30559 Hannover, Germany

**Keywords:** Alpaca, Neoplasia, Gastric ulcer, Adenocarcinoma, Squamous cell carcinoma, Animal physiology, Gastric cancer

## Abstract

In pathological examinations, gastric ulcers are often detected in South American camelids. The aetiology cannot be clarified in every case. However, tumour-related gastric ulcers are observed repeatedly. This study reports clinical, laboratory and pathological findings in six alpacas, three each with adenocarcinoma- and squamous cell carcinoma-associated gastric ulcers. Clinically they were presented with general symptoms like emaciation, anorexia and recumbency. Laboratory findings of these animals were non-specific. All animals were at least nine years old. The adenocarcinomas as well as the squamous cell carcinomas were metastasised in various organs, especially in the lymph nodes. Two adenocarcinoma-associated gastric ulcers were perforated. In summary, squamous cell carcinomas and adenocarcinomas can lead to ulcerative lesions in the gastrointestinal tract. Although neoplasms are rare overall, they should be considered as a possible differential diagnosis in the case of non-specific symptoms. In particular, older animals seem to be affected more frequently.

## Introduction

Gastric ulcers are a common finding in South American camelids (SACs; alpacas and llamas), but a definite clinical diagnosis is usually difficult^1^. A recent evaluation of 187 necropsy reports of alpacas from the Clinic for Swine and Small Ruminants, University of Veterinary Medicine Hannover, Germany revealed that a total of 23.5% of the animals had gastric ulcerations, with the third compartment (C3) being most frequently affected^2^. Previously, Theuß et al.^3^ analysed 233 necropsy reports of alpacas from Germany and came to a similar conclusion: they detected inflammatory alterations of the compartments in 34% of the animals, most of which had an erosive to ulcerative character^3^. Additionally, there are data on the presence of gastric ulcers in SACs from other countries: in Sweden, the evaluation of 107 necropsy cases of alpacas by Björklund^4^ revealed gastric ulcers in 3.7% of the animals. Twomey et al.^5^ evaluated 1477 carcasses from SACs from England and Wales and found a prevalence for gastric ulcers of 5.3%. O'Conor Dowd^6^ reported a prevalence for third compartment ulcers of 6% in alpacas and 12% in llamas after examining 359 SACs from the USA. On the other hand, ulcerations of the oesophagus and/or C1 were found less frequently in this evaluation (in 2.5% of the alpacas and in 4.8% of the llamas)^6^. In another recent study from the USA, Clarke and Breuer^7^ found ulcerative gastritis in five out of a total of 154 SACs (3.2%). After evaluating 93 SACs from Canada, Shapiro et al.^8^ also concluded, without giving exact numbers, that gastric ulcers are a common diagnosis.

Although inflammatory changes can occur in all three compartments in SACs, it is noticeable that ulcerations are most commonly found in the distal part of the third compartment (C3)^1,3,5^. This is due to the different pH values in this part of the stomach; while the proximal part has a pH of 6–7, the distal part of the C3 has a pH of only about 2^9^. Factors that are likely to promote gastric ulcers in SACs include non-steroidal anti-inflammatory drug (NSAID) administration as well as various stress factors^4,9^. Stress can be caused, for example, by other previous illnesses of the animal, but social stress factors also play a major role in llamas and alpacas^9^. Due to stress, there may be an increase in gastric acid and pepsin and a decrease in prostaglandin E (PGE)^1^, which can lead to the destruction of the integrity of the gastric mucosa. There are also descriptions that neoplasia may contribute to the destruction of the gastric mucosa in older alpacas and llamas^10,11^.

During evaluation of the necropsy reports from the Clinic for Swine and Small Ruminants, University of Veterinary Medicine Hannover, the authors noticed that several of the affected animals had suffered from neoplasms of the compartments^2^. These were three cases with squamous cell carcinomas (SCC; alpacas A, B, C) and three cases with adenocarcinomas (AC; alpacas D, E, F). As previous descriptions of neoplasia in the compartments in SACs include detailed pathological descriptions, but little information on clinical and laboratory diagnostic parameters of affected animals^10,12,13^, this study will present clinical, laboratory and necropsy findings in these six alpacas.

## Material and methods

Necropsy reports of 223 SACs from the Clinic for Swine and Small Ruminants, Forensic Medicine and Ambulatory Service of the University of Veterinary Medicine Hannover, Germany from January 2005 until the end of November 2021 were evaluated. These findings were summarised recently^2,14^. For the evaluation in the present study, further data of the animals were also taken into account; the age of the animal given in full years according to the information provided by the owners, the bodyweight in kilogrammes (kg) after weighing at initial examination or before necropsy, the symptoms observed in the animal by the owners and information on the diet and herd size.

Clinical examination was performed according to the standard protocol for examination of SACs used at the clinic. This included the assessment of the animal's behaviour, the measurement of the rectal body temperature, the auscultation of the heart with counting of the heart rate, the bilateral auscultation of the lungs and assessing of the respiratory rate, auscultation of the contractions of the forestomach in the left *Fossa paralumbalis* for two minutes, the assessment of the Body Condition Score (BCS)^15^, the assessment of the FAMACHA^©^-Score^16^, the palpation of lymph nodes (*Lymphonodi (Lnn.) mandibulares; Lnn. parotidei; Lnn. retropharyngei; Lnn. cervicales superficiales; Lnn. subiliaci*) for size, consistency, painfulness, movability and heating and the palpation of the abdominal wall for tension or painfulness.

The haematological parameters as well as the faecal samples were collected according to the previous study^2^. In addition, the biochemical parameters of the animals were included in the evaluation in the present study and these were measured according to the routine methods of the clinic laboratory^17,18^. For one animal (C), a cytological examination of ascitic fluid was also performed. For this purpose, a cytospin was prepared, which was also evaluated according to routine laboratory methods^19^.

The retrospective evaluation of the necropsy reports was performed according to the previous study^2^. In all six alpacas a full necropsy was performed at the Department of Pathology of the University of Veterinary Medicine Hannover, Germany.

For clinical or laboratory parameters where data were available for all six animals, statistical comparisons between SCC and AC were calculated using SAS (SAS Enterprise guide, version 7.1; SAS Institute Inc., Cary, NC, USA). Data were tested for normal distribution using the Shapiro–Wilk test. For normally distributed data (if *p*-value of Shapiro–Wilk test ≥ 0.05), mean and standard deviation were reported, and a t-test was performed for comparison. For non-normally distributed data (if *p*-value of Shapiro–Wilk test < 0.05), median and range were reported, and a Mann–Whitney U test was performed for comparison.

### Ethics statement

The study was approved by the Research Ethics Committee of the University of Veterinary Medicine Hannover, Germany under the Approval-code TiHo_EA_14_13-24, as it is compatible with the animal welfare guidelines of the University of Veterinary Medicine Hannover and with European and German animal welfare laws. All data used for this study were collected during clinical treatment and pathological examination and were obtained to diagnose the clinical case after the owners had given written consent. Data evaluation was performed retrospectively.

## Results

### Signalement

The alpacas were from six different farms in Northern and Western Germany. They were all females aged 9 to 20 years. The animals suffering from SCC were older and lighter than the animals suffering from AC; however, these differences were not statistically significant (Table 1). Detailed data of the six animals are given in the Supplementary Information (S1).

**Table 1:** Comparison of the findings of three alpacas with gastric squamous cell carcinoma (SCC) and three alpacas with gastric adenocarcinoma (AC).

Neoplasia	Gastric squamous cell carcinoma (SCC)		Gastric adenocarcinoma (AC)		*p*-value	Reference
	Mean (SD) or Median (min–max)	Below/within/above RI	Mean (SD) or Median (min–max)	Below/within/above RI		
Age [years]	15.0 (5.0)		9.0 (9.0–11.0)		0.18	
Bodyweight [kg]	45.2 (4.4)		61.5 (11.3)		0.08	
Body temperature [°C]	36.8 (1.5)	1/2/0	36.6 (1.7)	1/2/0	0.87	37.5–38.9
Heart rate [1/min]	67.3 (14.7)	1/2/0	104.0 (10.6)	0/0/3	0.02	60–90
Respiratory rate [1/min]	28.7 (4.2)	0/0/3	32.0 (10.6)	0/1/2	0.64	10–20
WBC [G/L]	51.5 (33.2)	0/1/2	14.4 (11.7)	1/0/2	0.14	8.0–16.0
Haemoglobin (Hb) [g/L]	91.7 (32.1)	2/1/0	115.0 (23.0)	1/2/0	0.36	110–161
PCV [L/L]	0.22 (0.08)	2/1/0	0.25 (0.04)	2/1/0	0.49	0.26–0.37
MCHC [g/L]	423.0 (19.7)	1/2/0	451.3 (30.0)	0/1/2	0.24	411–454
Lymphocytes [G/L]	1.7 (0.8)	1/2/0	0.7 (0.2)	3/0/0	0.11	1.1–5.2
PMN [G/L]	30.2 (19.6)	0/0/3	12.3 (10.5)	1/0/2	0.24	3.4–9.1
Band neutrophils [G/L]	15.1 (17.0)	0/0/3	1.0 (0.85)	0/0/3	0.29	0–0.1
Metamyelocytes [G/L]	2.7 (3.9)		0 (0)		0.35	
Myelocytes [G/L]	0 (0)		0 (0)		0.51	
Eosinophils [G/L]	0 (0)	3/0/0	0 (0–0.1)	3/0/0	0.51	0.8–3.4
Basophils [G/L]	0 (0)	0/3/0	0 (0)	0/3/0	0.51	0–0.2
Monocytes [G/L]	1.0 (0.4)	0/2/1	0.4 (0.5)	1/1/1	0.18	0.2–0.9
NLR	27.7 (28.6)	0/0/3	17.2 (14.5)	0/1/2	0.48	0.5–2.9^a)^
LMR	2.1 (1.7)		7.0 (7.5)		0.33	

Mean (SD) or median (min–max) as well as the respective number of animals that were below, within or above the respective reference interval (RI) are indicated. The *p*-value indicates the result of the t-test or the Mann–Whitney U test. Clinical references according to Fowler^1^, haematological references according to Hengrave Burri, et al.^59^, except for NLR^a)^, which is according to Hajduk^60^. *NLR* neutrophil-to-lymphocyte ratio, *LMR* lymphocyte-to-monocyte ratio.

### Anamnesis

The alpacas with SCC were presented to the clinic due to (A) movement disorders, difficulty in standing up and weakness; (B) emaciation and recumbency; (C) anorexia and colic. The animals with AC were presented due to (D) anorexia; (E) emaciation and weakness; (F) recumbency, apathy, anorexia, diarrhoea and melena. The alpacas came from herds of different sizes, ranging from two (F) to 20 (B) animals. Feeding was indicated as hay, pasture and alpaca concentrates (except for (B) that received cattle concentrate).

### Clinical examination

Behaviour was described as calm in all animals. All six alpacas were observed lying a lot or were even recumbent. The clinical examination revealed tachycardia (> 90 bpm) in all three animals with AC, whereas the heart rate in the animals with SCC was within or below the reference interval^20^. Except alpaca D, animals of both groups showed tachypnoea (> 20/min)^20^. Table 1 compares the clinical and haematological findings of the alpacas with SCC with those of the alpacas with AC.

All animals revealed a poor nutritional status. The conjunctivae of most of the animals showed physiological FAMACHA^©^-scores^16^. For alpaca F this was not assessed and in alpaca D the conjunctivae were described as “pale”. Palpation of the abdominal wall revealed pain in two alpacas with AC (E and F). Palpation of the lymph nodes did not show any deviation from the norm in any of the animals with SCC and AC, and none of the animals showed colic symptoms during the examination.

Detailed results of the clinical examination are displayed in the Supplementary Information (S2).

### Haematology

Haematological examination revealed anaemia in two animals suffering from SCC (B, C) and two animals suffering from AC (D, F) as well as leukocytosis in the same animals (B, C, D, F) (Table 1). Neutrophilia with left shift was observed in all animals except alpaca E. This animal (E) with AC revealed leukopaenia with lymphocytopaenia, neutropaenia and increased band neutrophils. Lymphocytes were decreased in one alpaca suffering from SCC (A) and all alpacas suffering from AC (D, E, F). Monocytosis was found in one animal suffering from SCC (C) and one suffering from AC (D). All but one animal (E) showed a significantly increased neutrophil-to-lymphocyte ratio (NLR). However, alpaca E showed an increased lymphocyte-to-monocyte ratio (LMR) compared to the others.

Reticulocytes as a parameter for regeneration were determined in two animals with the lowest packed cell volume—PCV (B, C) and indicated moderate regeneration at 78/1000 (B) and 42/1000 (C) using data from dogs for orientation^21^.

Detailed haematological results for the individual animals can be found in the Supplementary Information (S3).

### Biochemistry

The biochemical parameters of the animals were not analysed uniformly; for most parameters data were available for only a few animals. The biochemical results for the individual animals are displayed in the Supplementary Information (S3).

Main findings were hypoproteinaemia in alpaca A with SCC and alpacas D and F with AC. Alpacas A and B with SCC as well as alpaca F with AC revealed hypoalbuminaemia. At least alpaca B with SCC and alpaca F with AC showed further evidence of liver damage by hyperbilirubinaemia and elevated plasma activities for GLDH, ASAT and AP. Hyperglycaemia was observed in all animals with AC. Animals with AC (D, E, F) were all hypocalcaemic. Copper and selenium were each determined in two animals with SCC (A, B) and one animal with AC (E). All animals had normal plasma selenium levels but elevated copper levels^22^.

### Faecal samples

One animal with SCC (C) showed a medium degree of infestation with gastrointestinal nematodes, while the other examined animals (A, B, D, E, F) showed a low degree of infestation with gastrointestinal nematodes. Faecal occult blood was tested in three animals (C, E, F); only one animal suffering from AC (F) revealed a positive result.

### Further development

#### Alpaca A with SCC

The alpaca refused feed intake and was recumbent after three days. It lost 2 kg of body weight within four days. Symptomatic treatment with antibiotics (tetracycline), NSAIDs (meloxicam), dexamethasone and infusions of nutritional solutions did not improve the condition. The animal was euthanised after five days by intravenous administration (*V. jugularis*) of pentobarbital (100 mg/kg bodyweight Euthadorm^®^ 500 mg/mL CP-Pharma Handelsgesellschaft mbH, Burgdorf, Germany).

#### Alpaca B with SCC

The alpaca refused feed intake. It could initially stand up with help and then walk independently but became recumbent after two days. After infusions of glucose solutions, the initially decreased body temperature increased to 37.8 °C. The animal received symptomatic treatment with antibiotics (amoxicillin), dexamethasone and infusions of nutritional solutions. In addition, ruminal fluid from a donor cow was administered orally. Since the condition of the animal deteriorated, it was euthanised after two days by intravenous administration (*V. jugularis*) of pentobarbital (114 mg/kg bodyweight Euthadorm^®^ 500 mg/mL CP-Pharma Handelsgesellschaft mbH, Burgdorf, Germany).

#### Alpaca C with SCC

The animal showed reduced feed intake, and its general condition worsened in the days following its admission to the clinic. After one day the animal had watery diarrhoea. In the following days, the animal was more often found in lateral recumbency. It was then able to stand up by itself, but with difficulty, and usually lay down again quickly. Treatment with antibiotics (amoxicillin), spasmolytics (butylscopolamine, metamizole), omeprazole, iron dextran and a phosphorus supplement did not have any effects.

**Fig. 1:**
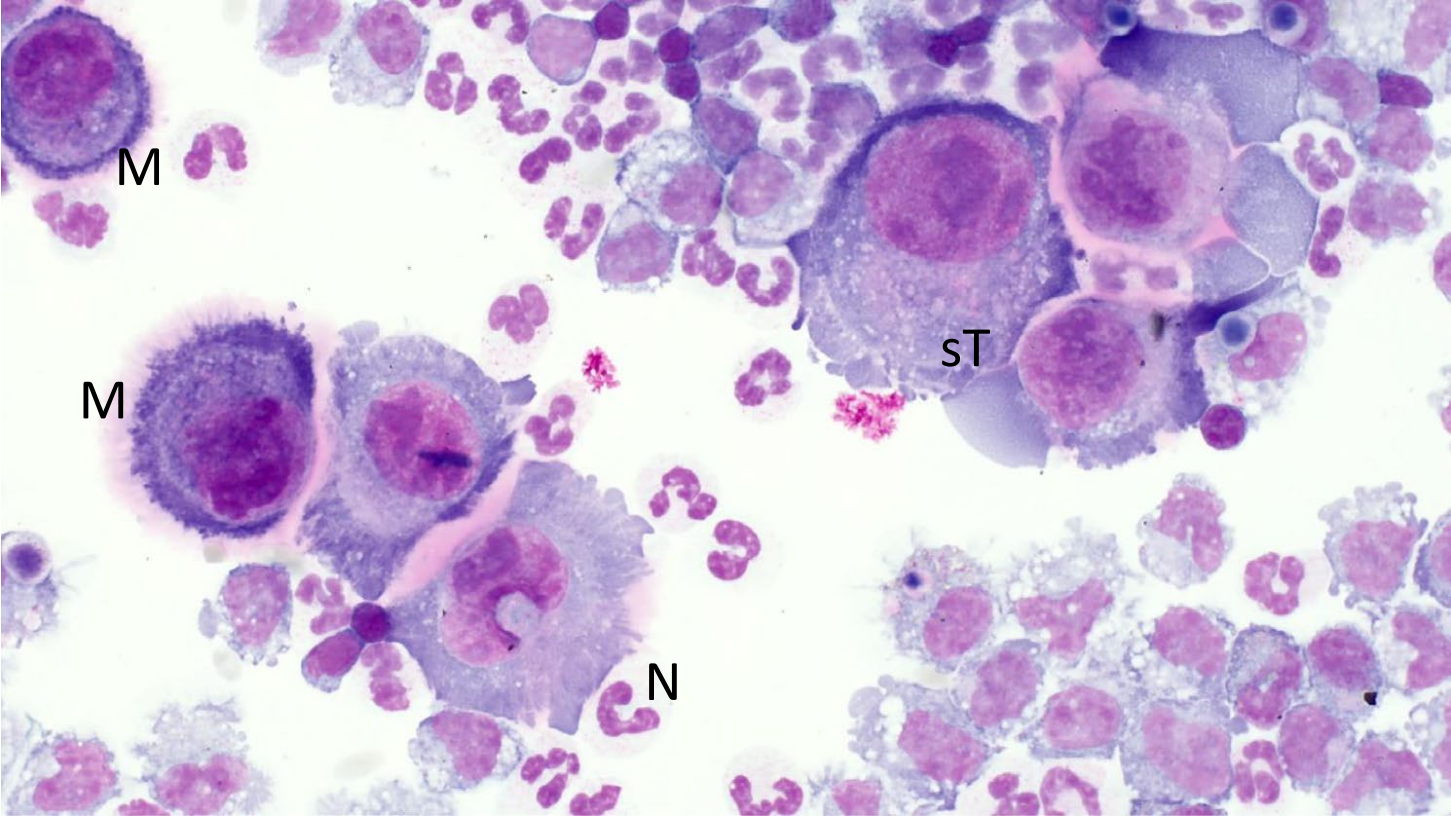
Cytology of the peritoneal fluid from alpaca C: activated mesothelial cells (M), increased band neutrophils (N) and increased protein gave a hint of an inflammatory process; suspected tumour cells (sT) indicated a neoplasia in the abdominal cavity. The suspected tumour cell is characterised by multiple irregularly shaped distinct nucleoli with severe anisonucleolosis. However, as mesothelial cells also change during inflammatory processes, it is difficult to reliably detect malignant alterations. Since the classification of tumour cells in cytological samples lacking the typical tissue architecture is not always easy, histological confirmation of a biopsy or at necropsy should be performed for confirmation.

Two ultrasound examinations of the abdomen were conducted. The first one took place at day three; no free fluid was detected in the abdomen, the uterus as well as the visible parts of the intestine were unremarkable. At the second examination at day five, fluid and fibrin-like structures were found in the abdominal cavity. This was punctured under ultrasound guidance by using a Veres needle. A cytological examination of the peritoneal fluid (Table 2) revealed high numbers of leukocytes and band neutrophils as well as suspected tumour cells, which could not be further differentiated microscopically (Fig. 1). However, the suspected neoplastic cell displayed multiple, distinct, irregularly shaped nucleoli and severe anisonucleolosis, which further raised the suspicion of a malignant transformation. In addition to the increased levels of leukocytes, the increased protein content also indicated an exudate. Based on the findings of the abdominal ultrasonography, peritonitis was diagnosed, and the animal was euthanised due to the poor prognosis by intravenous administration (*V. jugularis*) of pentobarbital (96 mg/kg bodyweight Euthadorm^®^ 500 mg/mL CP-Pharma Handelsgesellschaft mbH, Burgdorf, Germany).

**Table 2:** Laboratory results of the peritoneal fluid of alpaca C. References according to Cebra et al.^61^.

Parameter	Unit	Reference	Result
Leukocytes	G/L	0.25–1.89	2.16
Erythrocytes	G/L		0.25
Lymphocytes	%	0–4	8
PMN	%	41–85	27.5
Band neutrophils	%		23.5
Eosinophils	%	0	0
Basophils	%		0
Macrophages	%	14–55	41
Tumour cells			Not quantified
Protein	g/L	< 10–14	27.2
Albumin	g/L		9.3
Globulin/Albumin			1.92
Creatinine	µmol/L		72
Urea	mmol/L		8.9
Sodium	mmol/L	147 ± 3	142.5
Potassium	mmol/L	4.4 ± 0.6	4.5

#### Alpaca D with AC

On the day of admission, the animal had a premature birth (8–9 month gestation period); the cria died shortly after birth. One day later, an ultrasonography of the abdomen was conducted. There was no evidence of ascites. The animal refused feed intake, nutrients were given intravenously and orally. Symptomatic treatment with antibiotics (amoxicillin), NSAIDs (flunixin meglumine), spasmolytics (butylscopolamine, metamizole), infusions of nutritional solutions and dexamethasone was also performed. However, the animal’s condition deteriorated, and it died after 13 days.

#### Alpaca E with AC

The condition of the animal deteriorated further after admission to the clinic, despite the treatment with antibiotics (amoxicillin), spasmolytics (butylscopolamine, metamizole), infusions of nutritional solutions, omeprazole and dexamethasone. The alpaca was euthanised in agony about six hours after admission to the clinic by intravenous administration (*V. jugularis*) of pentobarbital (120 mg/kg bodyweight Euthadorm^®^ 500 mg/mL CP-Pharma Handelsgesellschaft mbH, Burgdorf, Germany).

#### Alpaca F with AC

The animal refused feed intake; symptomatic treatment with antibiotics (amoxicillin), NSAIDs (meloxicam), omeprazole, dexamethasone as well as nutrient preparations was performed. However, the animal’s condition continued to deteriorate, and the alpaca died after four days.

An examination for 25-OH-vitamin D further revealed that the alpaca was also suffering from vitamin D- deficiency (25-OH-Vitamin D: 29.8 nmol/L; reference value of the laboratory (SYNLAB Vet GmbH, Markkleeberg, Germany) for alpacas: 62.5–500 nmol/L).

### Pathology

At necropsy, all animals were in a reduced nutritional state with animals A, B and F being cachectic.

**Fig. 2:**
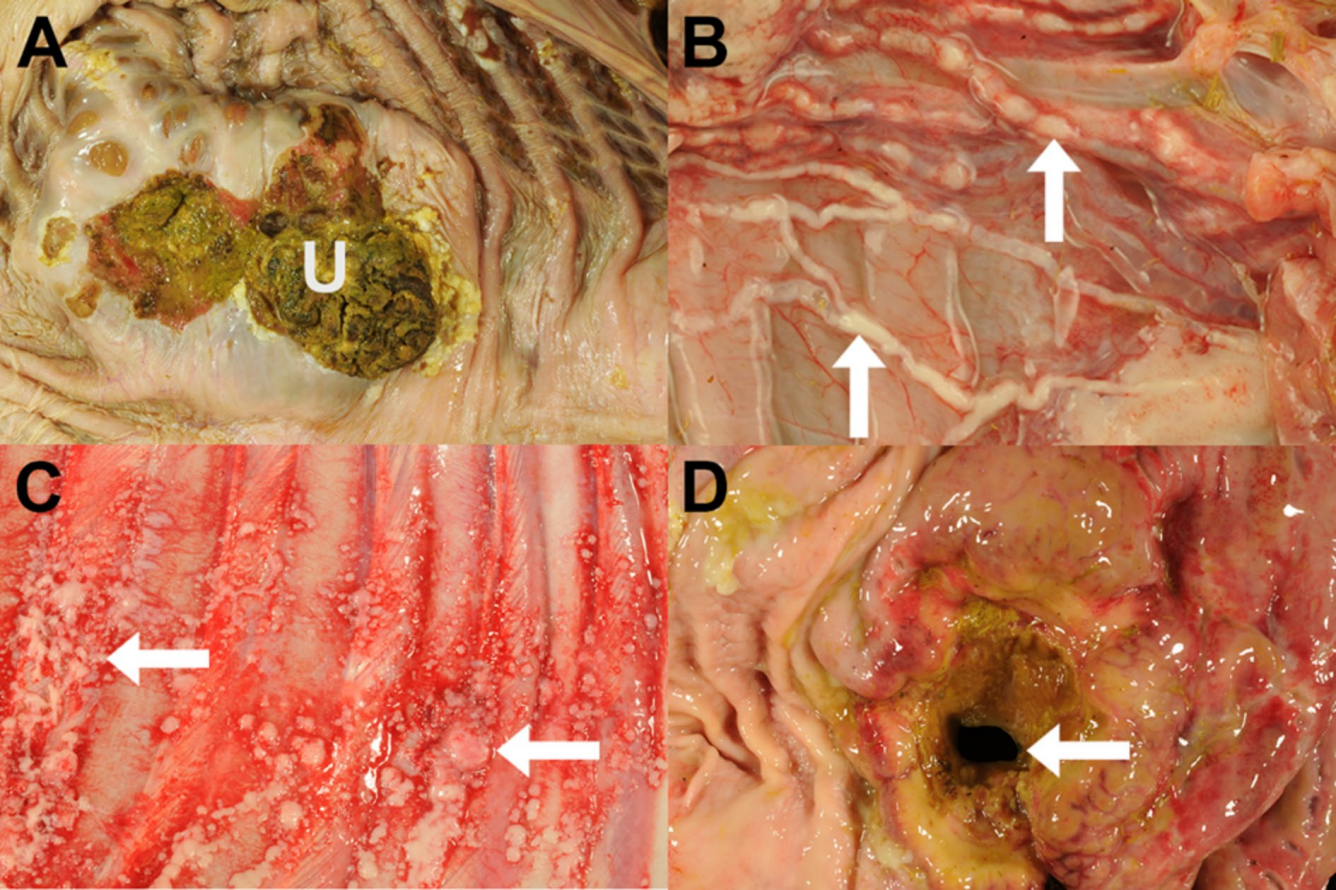
Macroscopical appearance of the neoplasms. (**A**) Ulcerated (U) squamous cell carcinoma within the first gastric compartment of an alpaca (alpaca C). (**B**) Severe lymphangitis carcinomatosa (arrows) in an alpaca with a squamous cell carcinoma of the first gastric compartment (alpaca A). (**C**) Pleural implantation metastasis (arrows) in an alpaca with a squamous cell carcinoma of the first gastric compartment (alpaca B). (**D**) Ulcerated adenocarcinoma within the third gastric compartment of an alpaca with perforation (arrow) of the gastric wall (alpaca E).

Macroscopically, alpaca A showed multiple white, firm masses with a diameter of 5 cm and multifocal chronic ulcerations in the first gastric compartment (C1). Histologically a gastric squamous cell carcinoma with lymphangitis carcinomatosa (Fig. 2B) and metastases in the lungs as well as in the pulmonary and mesenteric lymph nodes was diagnosed.

In addition, the animal showed a mild fibrinous peritonitis with 2 L serosanguinous intra-abdominal fluid, a mild multifocal, lymphoplasmacellular hepatitis and mild hyperostosis at the cervical vertebrae 6 and 7.

Alpaca B displayed a large (9 × 4 × 4 cm), white, firm, ulcerated mass with a central cavern filled with brownish serous fluid at the junction between gastric compartment 1 and 2 (C1/C2). Additionally, C1 showed an ulcer with a diameter of 6 cm with a raised rim. Histologically, the mass was characterised as a SCC with lymphangitis carcinomatosa. The animal displayed multiple metastases in lymph nodes (gastric, mesenteric, sternal, hepatic, pulmonary), the diaphragm, pleura (Fig. 2C), lungs, pericardium and liver.

Furthermore, the alpaca displayed mild to moderate multifocal myocardial fibroses, moderate multifocal, mostly suppurative pneumonia with intralesional bacteria and moderate multifocal, mostly suppurative hepatitis with necrosis.

**Fig. 3:**
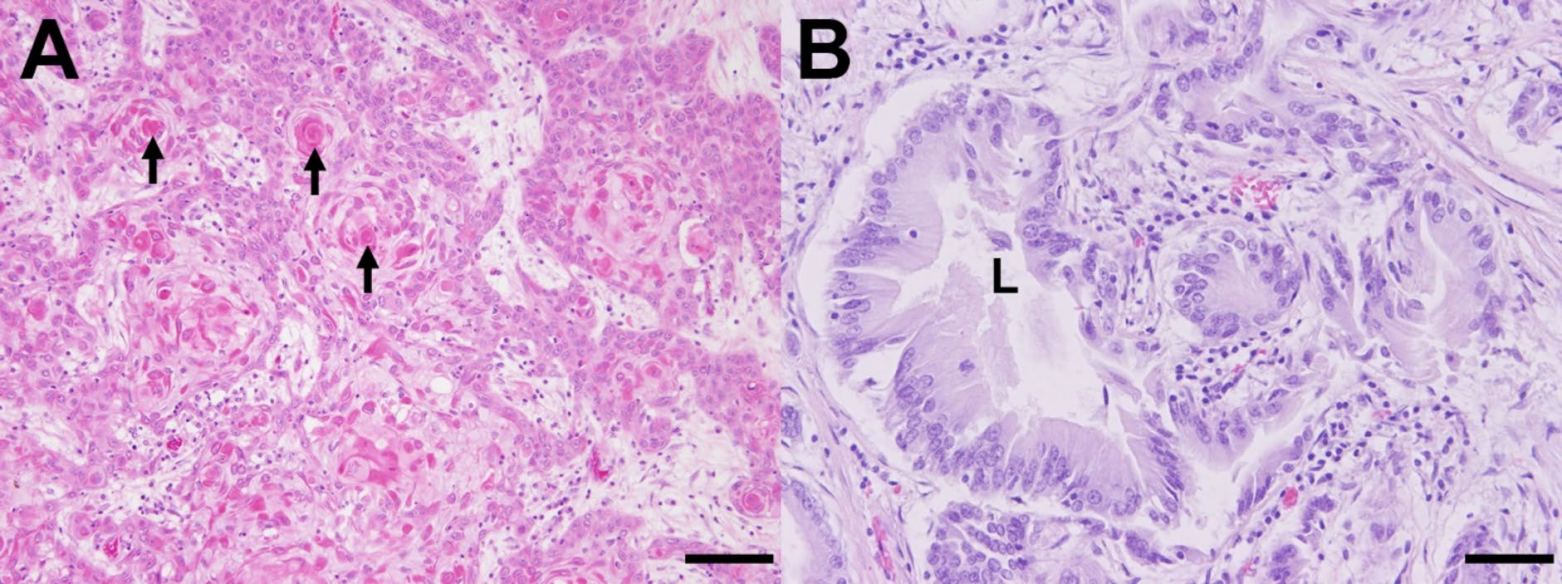
Histological appearance of the neoplasms. (**A**) Gastric squamous cell carcinoma in an alpaca. Neoplastic cells are forming nests and islands often with central keratinisation (arrows). Haematoxylin eosin stain. Bar = 50 µm (alpaca C). (**B**) Gastric adenocarcinoma in an alpaca. Neoplastic cells are forming irregularly shaped tubular and acinar structures forming a lumen (L). Haematoxylin eosin stain. Bar = 50 µm (alpaca E).

Alpaca C showed an infiltratively growing, multinodular, ulcerated mass (Fig. 2A), measuring 7 × 5 × 5 cm involving the first and second gastric compartment (C1, C2) and invading the pancreas, the duodenum and the colon from the serosal surface. Histologically, an ulcerated squamous cell carcinoma (Fig. 3A) with lymphangitis carcinomatosa and metastasis to the pulmonary lymph nodes and the diaphragm was present.

Additionally, a pericardial haemangioma, mild multifocal, suppurative pneumonia and hepatitis and multiple erosions and ulcerations of the third gastric compartment (C3) were observed.

At necropsy, alpaca D exhibited an ulcerated mass with a diameter of 10 cm and a focal thickening of the gastric wall and an adjacent, pedunculated, exophytic mass measuring 2 cm in diameter in the first gastric compartment (C1). Histologically, the mass was diagnosed as an invasive gastric adenocarcinoma with metastases in the gastric and mesenteric lymph nodes, the mesenterium and the peritoneum.

Furthermore, the alpaca showed a severe multifocal, erosive and ulcerative, transmural inflammation of the third gastric compartment (C3) with vasculitis and intralesional fungal hyphae. Similarly, a locally extensive, severe, necrotising endometritis with intralesional fungal hyphae was present. In addition, 20 L of a serosanguinous abdominal fluid was found.

Alpaca E showed a focal, transmural ulceration of the third gastric compartment (C3) with a diameter of approximately 1 cm. The surrounding gastric wall was thickened and firm over an area of 10–20 cm (Fig. 2D) and there was a fibrinopurulent peritonitis with 800 mL exudate. Histologically, a partly scirrhous adenocarcinoma of the third gastric compartment (Fig. 3B) with transmural ulceration and metastasis to the liver was found.

In addition, the animal was pregnant and a moderate fibrinosuppurative placentitis was diagnosed.

Alpaca F displayed a focal, transmural ulceration (2 cm in diameter) of the third gastric compartment (C3) with multiple adhesions of the gastric compartments with adjacent areas of the liver and abdominal wall. Furthermore, in this area a 14 cm round, cavernous, dark red mass and oligofocal white masses with a diameter of approximately 1 cm to 1.5 × 2 × 2 cm were found. Histologically an infiltratively growing, ulcerated adenocarcinoma with haemorrhages of the third gastric compartment with metastases to regional lymph nodes, hepatic lymph nodes, liver, mesenterium and serosal surface of the large intestine was diagnosed.

Furthermore, the animal exhibited severe diffuse necrosuppurative rhinitis with intralesional fungal hyphae, moderate multifocal, necrosuppurative bronchiolitis and a thyroid adenoma.

A detailed overview of all clinically relevant pathological findings and findings of minor or questionable clinical relevance are given in the Supplementary Information (S4).

## Discussion

As clinical and laboratory diagnostic findings in SACs with gastric ulcers are usually non-specific, they are often only detected in pathological examinations^2^. The aetiology can usually not be clarified, but in general, stress is considered to be the main cause of gastric ulcers. In addition to stress, tumour-associated gastric ulcers also seem to be a factor. In the present study, six animals with gastric ulcers associated with squamous cell carcinoma (SCC, *n* = 3) and adenocarcinoma (AC, *n* = 3) were further investigated.

Neoplasms are frequently found in post-mortem examinations of SACs. Valentine and Martin^23^ investigated the prevalence of neoplastic diseases in 551 camelids (368 alpacas and 180 llamas). The prevalence of neoplasia in llamas (11%) was higher than in alpacas (4.9%). Other authors mention prevalences between 3% and 8.8%^3–7,12^ in SACs. Malignant round cell tumours, especially lymphomas, seem to be very common^12,24,25^. However, squamous cell carcinomas and adenocarcinomas are also detected occasionally^12,23^.

According to Aboellail et al.^12^ there is a female predisposition for the occurrence of neoplasms. The authors assumed that this might be related to the relative frequency of mammary and uterine tumours, but also found a female predisposition for non-genital tumours. Consistent with these findings, only females were affected in the present study. Although the sex distribution in the 223 necropsy reports was not completely even, only a few more females (56.1%) than males (43.9%) were necropsied during the study period^14^. Age also seems to be a risk factor for tumours. It is described that, apart from lymphomas, older animals are more likely to be affected by neoplasia^12^, which is also consistent with the results of the present cases.

Squamous cell carcinoma (SCC) appears to be the most common malignant epithelial tumour in SACs^12^. Nevertheless, there are only a few reports of SCC in the stomach of SACs; predominantly C1 seems to be affected^10,13,26^. In accordance with the descriptions in those reports, a clearly circumscribed ulcerated lesion of the stomach wall of C1, partly C2, was also found in the three alpacas examined in the present study. Nonetheless, SCC can also occur without mucosal lesions inside the stomach; Rosiers et al.^11^ describe an SCC in C1 in an alpaca as a solid mass of the dorsal stomach wall which extended towards the aorta without mucosal changes in C1. While C3 in SACs is exclusively lined with glandular mucosa, in C1 and C2, in addition to areas with glandular mucosa, there are also areas with stratified squamous epithelium^27^. In this respect, it is to be expected that this type of tumour is usually observed in the first two compartments. However, Sartin et al.^10^ were even able to detect an SCC originating from the glandular mucosa in C3 in a llama. The authors consider metaplasia of the glandular epithelium, growth of heterotopic squamous cell debris or growth of the crypt gland base from multipotential cells as possible causes of a squamous cell carcinoma arising from glandular mucosa. Environmental influences and genetic predisposition due to the small gene pool of this species were suspected as potential factors for the occurrence of SCCs. In the literature there are also reports of SCC in the stomach of other livestock species, such as horses^28–31^, sheep^32^ and cattle^33^. In addition to SCC in the stomach, there are also some reports of SACs with SCC of the toe^34^, oral SCC^35^, SCC of the sternal skin^36^ or SCC secondary to a traumatic wound^37^. Valentine and Martin^23^ reported SCC originating from hairy skin as well as of perineal and ocular tissue.

Adenocarcinomas (AC), especially adenocarcinomas of the gastrointestinal tract, are described in the literature as very aggressive tumours that disseminate quickly, whereas SCC are less likely to metastasise^12^. In the present study, no difference was observed between the two tumour types, and metastases to other organs or organ systems were found in all six alpacas. Other case reports have already reported metastatic SCC^11,26^. Nonetheless, since it remains unclear in the presented cases how quickly the tumours developed and when metastases formed during the disease progression, no statement can be made about the comparative aggressiveness of the two tumour types found here.

To the authors’ knowledge, there is only one report of an AC in the stomach of SACs^12^, but no further information is available on this animal. Instead, various authors report intestinal-^38,39^, biliary-^23^, oviductal-^40^, pulmonary-^36,41–43^ and mammary gland-adenocarcinomas^44^ in SACs. Furthermore, the literature contains a case of a gastric adenocarcinoma and its diagnosis in an Old World camel^45,46^ as well as case reports of a cow^47^ and a wapiti (*Cervus elaphus nelsoni*)^48^.

The fungal infection of the stomach wall in one alpaca with gastric adenocarcinoma found in the present study (D) must be regarded as a secondary event. It is described that gastric ulcers are considered as a portal of entry for fungal hyphae^49,50^.

Clinically, all six alpacas showed mainly non-specific symptoms such as anorexia, emaciation and recumbency. Other studies also reported the same non-specific symptoms in animals with gastric tumours^10,26^. Although five out of the six alpacas were found to have metastases in the lymph nodes post mortem, these metastases were always in the internal lymph nodes. However, in none of the cases were there enlargements in the palpable external lymph nodes, whereas enlarged lymph nodes or a pathological mass can often be palpated in camelids with malignant lymphomas^25^. Also, the abdomen was painful in only two alpacas, and these were the two animals in which the gastric wall was finally perforated by the tumour (E, F). In the haematological and blood chemistry examinations, several pathological findings could be detected, but these were also non-specific. Contrary to the assumption that leukocytosis could be expected in the six animals due to the inflammatory processes in the stomach, there was also one animal with physiological leukocyte values (A) and one animal with leukopaenia (E). A possible explanation for these findings could be the increased consumption of leukocytes, which exceeds the compensatory capacity of the bone marrow in these animals^51^. Neutrophilia was observed in five of the animals (A-D, F), suggesting inflammatory processes probably due to tumour-related tissue damage. Increased levels of band neutrophils were found in all six animals, but such findings are also described in animals with lymphomas^25^ and animals with gastric ulcers as well as other wasting diseases^2^. A markedly increased neutrophil-to-lymphocyte ratio (NLR) was found in five of the animals (A-D, F). Little is known about the NLR or LMR in alpacas, but these parameters are used in other species as a prognostic marker in various tumour diseases including AC and SCC. An increased NLR^52–54^ or a decreased LMR^55,56^ is associated with a poorer prognosis. Reference values for LMR in alpacas are not yet available. Nonetheless, in human medicine it is around 4 to 6 in healthy individuals^57,58^. On this basis, the LMRs of three animals would be significantly reduced (A, C, D).

Some pathologically altered biochemical parameters can also indicate a disruption of specific tissue functions; Cebra et al.^25^ noted occasionally altered values in animals with malignant lymphoma. In the present study, two out of three animals with metastases in the liver also had increased levels of ASAT and GLDH.

Clinical, sonographic, radiographic and cytological examinations of puncture specimens or biopsies can help to indicate the presence of tumours^25,43,46^. However, a definite diagnosis is often difficult in a living animal. Only in alpaca C did the examination of the ascitic fluid reveal blastoid cells, thus indicating a tumorous disease. Endoscopy is not a reliable diagnostic tool for the detection of gastric tumours. Due to the anatomy of the camelid´s stomach, the examination of C3 is not possible^9,27^. The C1 is usually filled with a lot of content, similar to ruminants. To obtain significant results from endoscopy, the animals need to fast for an extended period of time to reduce the feed content in the stomach; therefore, gastric tumours are usually diagnosed only post-mortem. However, in the case of an animal that was already starving at the time of presentation due to illness, an endoscopic examination of the stomach could be useful to confirm the diagnosis if a gastric tumour is suspected..

## Conclusion

Even though gastric tumours are very rare in SACs, they should be considered as a differential diagnosis for non-specific symptoms such as emaciation, anorexia and recumbency, especially in older animals. Ultrasound examinations of the abdomen may help to identify primary or metastatic tumours.

## Supplementary Information

Supplementary Information.

## Data Availability

The datasets generated in this study are included in this article and are also available from the corresponding author upon reasonable request.
