# Supplementary material for: Adenocarcinoma and squamous cell carcinoma associated with gastric ulcers in alpacas
Source: Sci Rep. 2024 Dec 23;14:30586. doi: 10.1038/s41598-024-71079-x (PMC11666737; doi:10.1038/s41598-024-71079-x)
Supplement: Supplementary file 1 — Supplementary Information. [file 41598_2024_71079_MOESM1_ESM.pdf]

Online Resource 1

Adenocarcinoma and squamous cell carcinoma associated with gastric ulcers in alpacas

Saskia Neubert (0000-0002-5602-6686)[1,*,†], Matthias Gerhard Wagener (0000-0003-3366-8579)[1,*,†], Martin Ganter (0000-0003-1589-0803)[1], Christina Puff (0000-0002-2592-6868)[2]

[1] Clinic for Swine and Small Ruminants, Forensic Medicine and Ambulatory Service, University of Veterinary Medicine Hannover, Foundation, 30173 Hannover, Germany

[2] Department of Pathology, University of Veterinary Medicine Hannover, Foundation, 30559 Hannover, Germany

[*] Correspondence: neubert.saskia@web.de; matthias.gerhard.wagener@tiho-hannover.de

[†] These authors contributed equally to the manuscript and share first authorship

S1 Signalement of the six evaluated alpacas.

Neoplasia	Gastric squamous cell carcinoma (SCC)			Gastric adenocarcinoma (AC)		
Alpaca	A	B	C	D	E	F
Year of presentation	2015	2016	2019	2007	2017	2021
Age (years)	15	10	20	11	9	9
Sex	female	female	female	female	female	female
Bodyweight (kg)	50.0	44.0	41.5	74.0	58.5	52.0

S2 Clinical parameters of the animals. References for body temperature, heart rate, respiratory rate, contractions C1 according to Fowler [1], reference for BCS according to Wagener and Ganter [2], reference for FAMACHA©-score according to Wagener et al. [3].

Neoplasia	Gastric squamous cell carcinoma (SCC)			Gastric adenocarcinoma (AC)			Reference
Alpaca	A	B	C	D	E	F	
Body temperature (°C)	37.6	35.1	37.7	37.5	34.6	37.6	37.5-38.9
Heart rate (bpm)	84	62	56	96	100	116	60-90
Respiratory rate /min	32	30	24	20	36	40	10-20
Contractions C1 / 2 min	3	2	4	0	ND	ND	3-4/min
BCS	2	1	2	"moderate"	1.5	1.5	3
Lymph nodes	normal	normal	normal	normal	normal	normal	
Colic	no	no	no	no	no	no	
FAMACHA©-score	1	2	2	"pale"	2	ND	1-2
Abdominal wall	normal	normal	normal	ND	tense, painful	painful	

BCS – Body Condition Score; ND – not determined.

S3 Laboratory findings from the animals. References according to Hengrave Burri et al. [4], except for NLR [a)], which is according to Hajduk [5] and for copper [b)] and selenium [b)], which are according to Stanitznig et al. [6].

NLR – neutrophil-to-lymphocyte ratio, LMR – lymphocyte-to-monocyte ratio.

Neoplasia	Gastric squamous cell carcinoma (SCC)			Gastric adenocarcinoma (AC)			Reference
Alpaca	A	B	C	D	E	F	
Haematology							
WBC [G/L]	13.5	65.9	75	24.2	1.4	17.6	8.0-16.0
Haemoglobin (Hb) [g/L]	125	89	61	115	138	92	110-161
PCV [L/L]	0.3	0.2	0.15	0.25	0.29	0.22	0.26-0.37
MCHC [g/L]	417	445	407	460	476	418	411-454
Lymphocytes [G/L]	1.01	2.64	1.5	0.73	0.46	0.88	1.1–5.2
PMN [G/L]	11.2	50.41	28.88	20.57	0.49	15.75	3.4–9.1
Band neutrophils [G/L]	0.4	11.2	33.75	1.94	0.36	0.79	0–0.1
Metamyelocytes [G/L]	0	0.99	7.12	0	0.01	0	
Myelocytes [G/L]	0	0	2.25	0	0	0	
Eosinophils [G/L]	0	0	0	0	0.05	0	0.8–3.4
Basophils [G/L]	0	0	0	0	0.01	0	0–0.2
Monocytes [G/L]	0.88	0.66	1.5	0.97	0.03	0.18	0.2–0.9
Reticulocytes [1/1000]	ND	78	42	ND	ND	ND	
NLR	11.5	23.7	48.0	30.8	1.9	18.8	0.5-2.9[a)]
LMR	1.15	4.00	1.00	0.75	15.33	4.89	
Biochemistry							
Bilirubin [µmol/L]	ND	1.8	ND	ND	ND	3.23	0.2–1.01
Total protein [g/L]	54	60.7	ND	54.5	ND	50.9	56.2–70.4
Albumin [g/L]	27	21.8	ND	ND	ND	25.3	28.4–37.4
CK [U/L]	49	1718	ND	16	69	180	43–276
ASAT [U/L]	162	433	ND	43	55	373	155–248
GLDH [U/L]	12	348	5	8.5	ND	71	4–21.2
AP [U/L]	ND	770	48	ND	ND	289	30–144
GGT [U/L]	ND	195	ND	ND	ND	ND	15–43
Creatinine [µmol/L]	60	161	ND	315	164	ND	104–168
Urea [mmol/L]	6.96	11.05	ND	12.2	5.32	ND	4.5–9.1
Glucose [mmol/L]	ND	3.31	ND	13.99	20.44	18.49	5.4–7.3
Calcium [mmol/L]	1.9	3.39	2.16	2.02	1.67	1.97	2.1–2.5
Magnesium [mmol/L]	0.88	ND	ND	0.94	0.99	0.76	0.8–1.1
Phosphate [mmol/L]	0.99	ND	0.97	3.96	ND	3.95	1.1–2.8
Sodium [mmol/L]	148	ND	ND	ND	ND	ND	148–155
Potassium [mmol/L]	3.9	ND	ND	ND	ND	ND	4–5.2
Copper [µmol/L]	16.2	24.9	ND	ND	12.9	ND	4.88-10.37[b)]
Selenium [µg/L]	190.9	164.5	ND	ND	148.3	ND	15.7-206.3[b)]

ND – not determined.

S4 Pathological findings of the evaluated alpacas. Findings of minor or questionable clinical relevance are marked in grey.

Neoplasia		Gastric squamous cell carcinoma (SCC)			Gastric adenocarcinoma (AC)		
Alpaca		A	B	C	D	E	F
Nutritional status		Cachexia	Cachexia	Poor	Moderate	Moderate	Cachexia
General diagnoses		-	-	-	Uraemia	-	-
Diagnoses in the respective organs/organ systems							
Cardiovascular system		-	Pericardium with tumour metastases (5 mm diameter); mild to moderate multifocal myocardial fibrosis	Pericardium with 0.5 cm diameter, mineralised haemangioma	-	-	Left ventricle with moderate myocardial necrosis with calcifications and acute haemorrhage
Haematopoietic system		Pulmonary and mesenteric lymph nodes with tumour metastases	Various lymph nodes with tumour metastases	Pulmonary lymph nodes with tumour metastases	Gastric and mesenteric lymph nodes with tumour metastases	-	Various lymph nodes with tumour metastases
Respiratory system		Subpleural tumour metastases	Lung with tumour metastases (0.5 cm diameter) and lymphangitis carcinomatosa; moderate multifocal, mostly suppurative, partly lymphocytic pneumonia with intralesional bacteria	Mild multifocal, suppurative pneumonia and rhinitis	-	Mild focal, purulent rhinitis; Mild multifocal, granulomatous pneumonia	Severe diffuse, necrotising and suppurative rhinitis with intralesional fungal hyphae; lung with moderate multifocal, necrosuppurative bronchiolitis and mild multifocal, subpleural fibrosis
Gastrointestinal tract	Mouth	-	-	-	-	-	Dental malocclusions
	Oesophagus	-	-	-	-	-	-
	Compartments	• Macroscopic: C1 with multiple 5 cm diameter white, firm masses; multifocal chronic ulcerations<br>• Histologically: C1 with invasive gastric squamous cell carcinoma with erosion and multifocal haemorrhages, lymphangitis carcinomatosa	• Macroscopic: C1 with a 6 cm diameter ulcer with a raised rim; serosa with multifocal, white, firm masses (3 cm diameter); between C1 and C2 a 9x4x4 cm white, firm mass with a cavern with brownish serous content<br>• Histologically: C1 with gastric squamous cell carcinoma	• Macroscopic: C1/C2 with 7x5x5 cm, multifocal ulcerated mass with involvement of pancreas and parts of duodenum and colon; C2/C3 with multifocal erosions and ulcerations<br>• Histologically: C1/C2 with ulcerated gastric squamous cell carcinoma with necrosis and lymphangitis carcinomatosa	• Macroscopic: C1 with a 10 cm diameter area with thickened gastric wall and diphteroid-necrotising inflammation and a 2 cm sized exophytic proliferation of the mucosa; C3 with a 4 cm diameter necrosis of the gastric wall and multifocal ulcerations in the glandular area<br>• Histologically: C1 with invasive gastric adenocarcinoma; C3 with a severe multifocal, erosive to ulcerative, partly necrotising transmural gastritis and detection of fungal hyphae	• Macroscopic: Caudal third of C3 with a 1 cm diameter perforated ulcer with a raised rim; 10-20 cm diameter circular expansion of the ulcer (inflamed and firm gastric wall)<br>• Histologically: C3 with an invasive, indurated adenocarcinoma with moderate multifocal, lymphohistiocytic gastritis; C2 with mild diffuse, lymphohistiocytic, partly eosinophilic gastritis	• Macroscopic: Caudal third of C3 with a 2 cm diameter perforated ulcer; adhesions of the compartments with liver and abdominal wall<br>• Histologically: C3 with an invasive adenocarcinoma with associated ulceration

	Intestine	-	-	Squamous cell carcinoma growing infiltratively in the small intestine with lymphangitis carcinomatosa; mild neutrophilic inflammation of the colon	-	Moderate lymphohistiocytic, partly neutrophilic, partly eosinophilic enteritis	-
Body cavities		Mild fibrinous peritonitis (2 L serous intra-abdominal fluid)	Diaphragm with tumour metastases (1 cm diameter); mild multifocal purulent inflammation of the diaphragm	Diaphragm with tumour metastases; 500 mL gelatinising intra-abdominal fluid	Mesentery and peritoneum with invasive adenocarcinoma (multiple, white nodules); moderate lymphoplasmacellular peritonitis (20 L serous intra-abdominal fluid)	Fibrino-purulent peritonitis with ingesta and bacteria (800 mL dirty intra-abdominal fluid)	Mesentery and colon serosa with tumour metastases; 14 cm diameter haematoma with tumour metastases; adhesions of organs; 2.5 L brownish serous intra-abdominal fluid; thyroid adenoma
Liver		Mild multifocal, lymphoplasmacellular infiltration	Multiple tumour metastases (3 cm diameter); moderate multifocal suppurative, partly lymphoplasmacellular hepatitis with necrosis	Mild multifocal, suppurative hepatitis	Moderate multifocal granulomatous hepatitis with hepatocellular necrosis	Tumour metastases; mild to moderate, purulent, partly lymphohistiocytic hepatitis	Tumour metastases
Genitourinary tract		-	Mild focal lymphoplasmahistiocytic nephritis	-	Severe necrotising endometritis with detection of fungal hyphae (previous abortion); bilateral tubulonephrosis (tubular necrosis and calcifications)	Moderate fibrinosuppurative placentitis; mild, lymphohistiocytic nephritis and cystitis; pregnant	Mild multifocal, interstitial fibrosis of the kidneys
Musculoskeletal system		Cervical vertebrae 6 and 7: mild hyperostosis	Skeletal muscles with mild muscle fibre degeneration and partly suppurative inflammation	-	-	-	-
Skin		-	Moderate diffuse orthokeratotic hyperkeratosis and follicular keratosis	Elbow with moderate diffuse orthokeratotic hyperkeratosis, follicular keratosis and dermal sclerosis	-	-	-
Nervous system		-	Rete mirabile: mild multifocal lymphohistiocytic vasculitis	Rete mirabile: mild multifocal lymphohistiocytic vasculitis	-	-	-
Eyes/Ears		-	Detached retina	-	-	-	-

References

1. Fowler, M. Physical examination, restraint and handling. *Vet. Clin. North Am. Food Anim. Pract.* **5**, 27-35, doi:10.1016/S0749-0720(15)31001-X (1989).
2. Wagener, M. G. & Ganter, M. Body Condition Scoring bei Neuweltkamelen [Body Condition Scoring in South American camelids]. *Prakt. Tierarzt* **101**, 684–696, doi:10.2376/0032-681x-2020 (2020).
3. Wagener, M., Meyer zu Westerhausen, M., Neubert, S. & Ganter, M. Identifizierung anämischer Alpakas und Lamas mithilfe des FAMACHA©-Scores [Identification of anaemic alpacas and llamas using the FAMACHA© score]. *Prakt. Tierarzt* **103**, 620-629, doi:10.2376/0032-681X-2227 (2022).
4. Hengrave Burri, I., Tschudi, P., Martig, J., Liesegang, A. & Meylan, M. Neuweltkameliden in der Schweiz. II. Referenzwerte für hämatologische und blutchemische Parameter [South American camelids in Switzerland. II. Reference values for blood parameters]. *Schweiz. Arch. Tierheilkd.* **147**, 335-343, doi:10.1024/0036-7281.147.08.335 (2005).
5. Hajduk, P. Haematological reference values for alpacas. *Aust. Vet. J.* **69**, 89-90, doi:10.1111/j.1751-0813.1992.tb15558.x (1992).
6. Stanitznig, A. *et al.* Hämatologische und blutchemische Parameter sowie Mineralstoff-und Spurenelementkonzentration im Serum bei Neuweltkamelen in Österreich [New World camelids in Austria: a compilation of haematological and clinical-chemical parameters as well as serum concentrations of minerals and trace elements]. *Wien. Tierarztl. Monatsschr.* **105**, 3-11 (2018).
